# Diagnostic Symptoms

**Published:** 1904-11

**Authors:** 


DIAGNOSTIC SYMPTOMS.

The diagnostic symptoms of pericemental abscess are distinct and plain when once distinguished. In acute cases the first manifestation is pain; not severe, but continuous, and located by the patient in the offending tooth. Nervous apprehension on the part of the patient is, I believe, always incident to it. The trouble is liable to be confounded with periodontitis, but is readily distinguished from it. In acute pericemental abscess, there are no marked inflammatory symptoms in the alveolar and gum tissue; no swelling, no acute pain in response to tapping or pressure, no marked distinction between hot and cold applications, and no relief afforded by any local medication. The loosening of the tooth, slight in the beginning, becomes more and more marked as the case progresses. It will thus be noted that while this affection has been, and is, liable to be confounded with periodontitis, all the diagnostic symptoms (except the pain and the response to pressure) in the former, are exactly the opposite to those in the latter.

Chronic pericemental abscess is distinguished by absence of pain and all other active inflammatory symptoms. Occasionally, there is some hypertrophy of the gum tissue, but no marked swelling, neither soreness of the tooth on pressure. The tooth itself can generally be used in mastication without special discomfort. The escaping pus is discharged at the edge of the alveolus—between the alveolus and the gum. The point of egress may be distinguishd by a peculiar lip, or mouth-like opening, marked by a circumscribed turgescent condition of the gum tissue; a condition wholly unlike the fistulous opening of an alveolar abscess.

